# Fabrication of microplastic and nanoplastic particles and fibres for use in pulmonary toxicity studies

**DOI:** 10.1186/s12989-025-00641-w

**Published:** 2025-11-06

**Authors:** Eric Auyang, Mengzheng Ouyang, Adam Laycock, Henry Blake, Teresa D. Tetley, Timothy W. Gant, Anne E. Willis, Stephanie Wright

**Affiliations:** 1 MRC Centre for Environmental and Health, Environmental Research Group, School of Public Health, Faculty of Medicine, Imperial College London, London, W12 0BZ UK; 2 Lung Cell Biology, Airways Disease, National Heart & Lung Institute, Imperial College London, London, W12 0HS UK; 3 Department of Earth Science and Engineering, Imperial College London, London, SW7 2AZ UK; 4 HPRU in Environmental Exposures and Health. Toxicology Department, Radiation, Chemical and Environmental Hazards Directorate, UK Health Security Agency, Harwell Campus, Oxfordshire, OX11 0RQ UK; 5 MRC Toxicology Unit, Cambridge University, Tennis Court Road, Cambridge, CB2 1QW UK

**Keywords:** Microplastics, Nanoplastics, Test materials, Microplastic fibres, Hazard assessment, Pulmonary toxicology

## Abstract

**Background:**

Micro/nanoplastics (MNPs) are a commonly detected environmental contaminant in indoor and outdoor environments. Airborne MNPs are of various shapes and sizes, some of which are small enough to reach the deep lung if inhaled. Current research into the toxicity of airborne MNPs in the lung has only involved a small number of polymers and shapes due to their limited availability. The most commonly available are polystyrene spheres and to date, these have been used in the majority of studies, though their relevance to environmental MNPs is limited. To address this gap, we aimed to develop a method to fabricate MNPs of three environmentally relevant polymers, producing both micro- and nano-sized particles as well as fibres. Enhancing the consistency and accessibility of test materials will enable researchers to better investigate how size, shape, and polymer type influence lung toxicity, while also reducing variability introduced during fabrication.

**Results:**

We successfully developed methods to fabricate MNPs of polyamide, polystyrene, and polyethylene terephthalate, as microplastics, nanoplastics, and fibres. MNPs were characterized for their chemical purity and size. The size of the fabricated MNPs were found to be of a respirable dimension. As a solvent-based method of preparation was used, leachates from the MNPs were analysed to check for contamination that could cause non-specific toxicity. These were found to have no effect on the metabolic activity of either THP-1 macrophages or transformed type-1 (TT1) epithelial cells.

**Conclusions:**

This work provides pulmonary toxicologists with a method for the fabrication of MNPs and their physical and chemical characteristics. Their characteristics indicate they are a representative test material for experimental systems.

**Supplementary Information:**

The online version contains supplementary material available at 10.1186/s12989-025-00641-w.

##  Introduction

Atmospheric micro/nanoplastics (MNPs) are a component of the solids and liquids that comprise airborne particulate matter (PM). A wide variety of polymer types and shapes have been reported in atmospheric sampling studies, the hazards of which are not well understood. The importance of understanding lung diseases caused by respirable, airborne particles < 2.5 μm is emphasised by the prominent cases of asbestos fibres, and coal or silica dust, the inhalation of which can lead to interstitial lung diseases [1]. The pathogeneses arising from the inhalation of these materials differ due to the different chemical and particle interactions both inside the alveoli and the interstitial space, specific to the physicochemical properties of each particle domain [2]. Airborne MNPs could trigger different pathways due to their own set of variable properties, which is why it is important that toxicity experiments cover several MNP types. In this study, we have fabricated MNPs with a size range suitable for pulmonary toxicity studies.

The presence of airborne MNP fragments and fibres has been reported in urban centres, remote areas, indoor and outdoor environments, using active and passive samplers [3–12], and their detection within fine particulate matter (PM_2.5_) samples poses a human health hazard should they penetrate the deep lung [13–15]. A range of polymers have been detected within these samples, with polypropylene (PP), polystyrene (PS), polyethylene (PE), polyamide (PA) and polyethylene terephthalate (PET) being represented in several studies [3–5, 10]. Fibres are a common shape, particularly within indoor environments, with PET being particularly prevalent due to its use in textiles, and PP, PE and PA having been detected to a lesser extent [3, 4, 13, 16, 17]. While textile fibres typically have a diameter in the tens of microns, fibrillation as a result of wear can produce MNP fibres of a respirable size range, which may pose a human health hazard [18–20].

The separation and isolation of respirable MNPs from PM_2.5_ samples is challenging, as the extraction process, typically via chemical digestion and density separation, could alter their physicochemical properties. Additionally, the amount of particulate matter required to generate enough MNP material for toxicology is substantial. Furthermore, it would be difficult to answer controlled questions about the biological effects of polymer type, size and shape, as extracted samples would be a mix of material. Therefore, the fabrication of MNPs is essential for advancing research on their environmental behaviour, biological interactions, and potential toxicity. Cryomilling and solvent-based precipitation techniques have gained prominence due to their capacity to produce MNPs from a range of feedstock materials. Cryomilling involves embrittling plastic feedstocks using liquid nitrogen before mechanically grinding them into smaller fragments. This approach is advantageous for its scalability and applicability to diverse polymer feedstocks, allowing for the generation of particle morphologies that mimic those found in environmental samples [21, 22]. However, cryomilling often yields a broad and unpredictable size distribution, which may require further size separation procedures such as sieving or settling to obtain particles within the desired size range [23].

Alternative solvent-based precipitation methods offer greater control over particle size and uniformity. In this technique, polymers such as polystyrene are first dissolved in a compatible solvent and then reprecipitated using an anti-solvent, resulting in the formation of MNPs [24]. These methods can be refined to produce spherical particles of defined diameters and have been employed effectively in both pharmaceutical and environmental contexts [25, 26]. Nonetheless, they often require the use of hazardous solvents, strict process controls, and post-treatment purification steps to ensure reproducibility and remove residual chemicals. Despite the available methods for fabricating MNPs of a suitable size for pulmonary toxicity studies, most in vitro and in vivo hazard studies to date have primarily used polystyrene (PS) nano (NP) or microplastics (MP) [27]. This is largely because these particles are widely commercially available. However, PS MPs synthesized commercially are usually near perfect pristine spheres as a result of emulsion polymerization, which are often coated in a surfactant to improve aqueous stability [28]. Wimmer et al. (2025) excellently summarises the currently available commercial MNPs for in vitro and in vivo research [29].

More recently, several papers have fabricated MNPs using polymers other than polystyrene, and in non-spherical shapes, contributing to the production of a more ‘environmentally relevant’ test material [23, 24, 27, 29–34]. However, current studies are limited by either the number of polymers included, or the size range being fabricated, and are rarely being made as fibrous particles. The fabrication of MNPs with a range of sizes, polymers, and shapes will allow researchers to fully explore the toxicity associated with MNPs and their physicochemical properties as experienced via environmental exposures. While environmental relevance is an important concept which helps to bridge the gap between laboratory results and real-world human exposures, the ability to fully recreate an environmental MNP, with all adsorbed chemicals, biomolecules and degrees of weathering is challenging because of their heterogeneity.

While not addressing all characteristics of the MNP, the methods presented here provide researchers with a methodology for the fabrication of some common environmental polymers, polyamide 6,6 (PA), PS, and polyethylene terephthalate (PET) as MPs, NPs, and fibres (MPFs) for pulmonary toxicity testing, collectively referred to herein as MNPs. These polymers have been chosen as they comprise prevalent environmental airborne MNPs [35]. Unlike previously published fabrication methods, we have used the same three polymer feedstocks to fabricate three different types of MNPs. The production method for these MNPs provides a resource for investigators to test the toxicological differences influenced by polymer type, size, and shape, for a more holistic approach to understanding drivers of MNP toxicity.

##  Materials and methods

###  Materials

####  Polymers

Polystyrene (PS) granules (Product No. 430102) were sourced from Sigma Aldrich, MA, USA, polyamide 6,6 (PA) (Product Code: AM30-GL-000100) were sourced from Goodfellow, Cambridge, UK, and ‘Reference Standard’ polyethylene terephthalate (PET) strips (Product No. 1546900) were sourced from the United States Pharmacopeia, MA, USA. Further information on polymer properties can be found on their manufacturer’s website.

#### Solvents

The list of solvents used for micro/nanoplastics (MNP) are shown (Table 1). Due to potential source to source variability, such as solvent purity, polymer additives, and polymer molecular weight, the specific product codes and supplier are included in Tables 1 and 2.

**Table 1:** List of solvents used in the fabrication process, product numbers, and suppliers

Solvent	Product no.	Supplier
N, N-dimethylformamide (DMF)	227,056	Sigma Aldrich, MA, USA
Formic acid (FA)	F0507	
1,1,1,3,3,3-hexfluoro-2-isopropanol (HFIP)	105,228	
Propan-2-ol	278,475	
Tetrahydrofuran (THF)	1,371,369,200	Thermo Fisher, MA, USA
Dichloromethane (DCM)	1,064,541,000	VWR Chemicals, PA, USA
Trifluoroacetic acid (TFA)	L06374.AE	
Ethanol	83813.360DP	

###  Methods

First, polymer solutions were prepared by dissolving the stock polymers in solvent. Table 2 outlines the solvents and concentrations for the fabrication of each type of MNP. Polymers will be referred to from here on as PA (polyamide 6,6,), PS (polystyrene), and PET (polyethylene terephthalate). All sonication was performed using a Soniprep 150 Plus probe sonicator. 10 μm stainless steel (SS) sieves were purchased from Endecotts (Derbyshire, UK).

**Table 2:** Polymers and their solvent concentrations for fabricating micro, nano, and fibrous plastics

Polymer	Particles			Fibres	
	Solvent	Concentration (mg/mL)		Solvent	Concentration (mg/mL)
		Micro	Nano		
Polyamide 6,6	Formic acid	40	10	Formic acid	250
Polystyrene	Tetrahydrofuran	10	5	N,N-dimethylformamide	250
Polyethylene Terephthalate	1,1,1,3,3,3-hexafluoro-2-isopropanol	25	20	Trifluoroacetic acid : Dichloromethane (70:30)	300

####  Glassware preparation

40 mL glass vials (Agilent Technologies, CA, USA), used for precipitation and 2 mL glass vials (Sigma Aldrich, MA, USA), used for long term storage of micro/nanoplastics (MNP), were washed prior to use. They were washed with a laboratory detergent and rinsed with deionized (DI) water. The vials were left to soak for 24 h in DI water with a small amount of detergent, followed by another rinsing with DI water. Vials and caps were then placed in a clean glass bottle, and sonicated in acetone for 5 min, then sonicated in methanol for another 5 min, following removal of the acetone. The vials and caps were then left to dry in a plastic-free drying cabinet. SS sieves were cleaned before and after each use in a sonicating water bath to remove particles stuck to the mesh.

####  Microplastic fabrication

*Micro-polystyrene* 2 mL of the PS solution (Table 2) was transferred to a 40 mL glass vial, and 20 mL of ethanol was quickly added under ultrasonication at 16 μm amplitude. The solution was then split into two separate 50 mL tubes containing 20 mL of ethanol each, to reduce the concentration of THF in solution prior to centrifugation.

*Micro-polyamide* 2 mL of the PA solution (Table 2) was transferred to a small glass vial with a PTFE coated stir bar. At room temperature, the solution was stirred at 2000 rpm for approximately 48 h, until most of the FA had evaporated, and the precipitated PA has a slush-like consistency. 5 mL of water was then added and left stirring at 7000 rpm for 1 h to disperse agglomerated particles.

*Micro-polyethylene terephthalate* 3 mL of PET solution (Table 2) was transferred to a 40 mL glass vial, and 2 mL of MiliQ water was added and gently mixed to initialize precipitation. A sonicating probe was then positioned directly above the surface of the solution, and 30 mL of ethanol was quickly added under ultrasonication at an amplitude of 16 μm.

##### Size separation and purification of microplastics

The fabrication method described in 2.2.2 can be performed multiple times, pooling an aggregate stock or batch, and processed using the following methodology. Residual solvent was removed through centrifugation and resuspension with ethanol. Precipitated MPs were transferred to a 50 mL tube and centrifuged at 3030 RCF for 30 min. The supernatant was removed, including any un-pelleted MPs, and replaced with another 20 mL of ethanol, and vortexed to resuspend the pellet. This was repeated 3 times, with the remaining ethanol dispersion being passed through a 10 μm SS sieve to remove larger particles, sieved MPs were transferred to a 50 mL tube. The mass of fabricated the MPs can be determined gravimetrically by allowing an aliquot of the solution to dry on a pre-weighed boat. A concentration /mL can be derived from the volume of the dried aliquot, and a total mass worked out based on the volume of suspended liquid.

The suspended MPs were centrifuged again for 30 min at 3030 RCF. The pellet was finally resuspended in 2-propanol and transferred to cleaned 2 mL glass vials and stored at -20 °C. The final resuspension volume can be adjusted as desired, to arrive at a suitable MP concentration for storage.

####  Nanoplastic fabrication

*Nano-polystyrene* 1 mL of PS solution (Table 2) was pipetted quickly into water heated to 50 °C and under ultrasonication at an amplitude of 16 μm. 5 mL of FBS was added immediately after to prevent aggregation, the mixture was then heated to 65 °C and stirred on a hotplate until the volume has reduced by half.

*Nano-polyamide* 2 mL of PA solution (Table 2) was pipetted dropwise into 30 mL of water under ultrasonication at an amplitude of 16 μm. Precipitation can often result in the formation of large particles which were removed by allowing the solution to settle for a few minutes before removing the supernatant and transferring it to a new vessel.

*Nano-polyethylene terephthalate* 2 mL of PET solution (Table 2) was transferred to a 40 mL glass vial, and a sonicating probe was positioned directly above the surface of the solution. 30 mL of water was then quickly added under ultrasonication at an amplitude of 16 μm. 5 mL of FBS was added immediately after to prevent aggregation, the mixture was then heated to 65 °C and stirred on a hotplate until the volume had reduced by half.

#####  Size separation and purification of nanoplastics

The fabrication method described in 2.2.2 can be performed multiple times and processed using the following methodology. Residual solvent was removed through washing and resuspension in FBS. Precipitated nanoplastics (NP) were transferred to a 50 mL tube and larger particles were allowed to settle for 30 min, before the supernatant was transferred to a new 50 mL tube. The suspensions were then aliquoted into 1.5 mL Eppendorf tubes and centrifuged at 21,100 RCF for 30 min. The supernatant was removed, and the pellets were resuspended in a 50% FBS: DI solution. The washing steps were repeated three more times. The mass of fabricated of NPs can be determined using the method stated in 2.2.2.1.

The suspended NPs were centrifuged again for 30 min at 21,100 RCF. Following the final centrifugation, the NPs were resuspended in the FBS: DI solution and transferred to cleaned 2 mL glass vials and stored at -20 °C. The final resuspension volume can be adjusted as desired, to arrive at a suitable NP concentration for storage. All work involving the use of FBS was performed in a sterile environment to prevent microbial contamination.

####  Microplastic fibre fabrication

A Bioinicia LE-50 was used for the electrospinning procedure, and Leica CM1950 cryostat was used for the cutting and production of microplastic fibres (MPF). The distance from the tip to the collector was 10 cm for all polymers, the collecting drum was rotating at 2,000 RPM. Concentrations and voltages were adapted from previous publications [36, 37].

*Polyamide fibres* PA was dissolved in 10 mL of 3:1 formic acid: dichloromethane at a 25% weight to volume ratio (w/v) (Table 2). Fibres were extruded at a rate of 0.5 mL/hour at 20 kV.

*Polystyrene fibres* PS was dissolved in 10 mL of dimethylformamide at a 25% w/v (Table 2). Fibres were extruded at a rate of 1 mL/hour at 20 kV.

*Polyethylene Terephthalate fibres* PET was dissolved in 10 mL of 70:30 trifluoracetic acid: dichloromethane at 30% w/v (Table 2). Fibres were extruded at a rate of 4.5 mL/hour at 15 kV.

##### Cryo-sectioning and size separation of microplastic fibres

Electrospun fibres were cut using a cryotome to achieve the desired lengths [38]. A small section of the electrospun fibre mat was first embedded in ice, excised, and mounted onto a cryotome stub using optimal cutting temperature medium (OCT). 8 μm sections were taken from the fibre mat, collected in a 50 mL tube, and resuspended in ethanol. The solution of cut MPFs was sonicated at 10 μm amplitude to disperse any tangled fibres. Any undispersed clumps were allowed to settle out of the solution, and the supernatant transferred to a new 50 mL tube. MPFs were washed with ethanol three times and weighed using the same method as the 2.2.2.1 and 2.2.3.1. Following the final centrifugation, the MPFs were resuspended in the 2-propanol and transferred to cleaned 2 mL glass vials and stored at -20 °C. The final resuspension volume can be adjusted as desired, to arrive at a suitable MPF concentration for storage.

####  Dynamic light scattering

Dynamic light scattering (DLS) for analysing MNP size distribution analysis was performed using a Malvern Panalytical Zetasizer Pro, with a helium-neon laser with a wavelength of 632.8 nm. Polystyrene cuvettes were used for size measurements, while folded-capillary cells were used for zeta-potential measurements. Samples were sonicated at 10 μm amplitude prior to analysis to maximize dispersion. Refractive index (R.I) for PA, PS and PET used for measurements were 1.53, 1.59 and 1.56, respectively.

####  Light microscopy

Light microscopy was performed to assist with MPF size measurements, using an Olympus BX53M and images were taken using an Olympus SC50 5-megapixel camera.

#### Fibre dimension measurements

Fibre dimensions were measured using a combination of scanning electron microscopy (SEM) and light microscopy images. Using ImageJ, the diameter of 50 fibres was measured using SEM images acquired using the methodology described below in 2.2.8. Fibre length was measured using images taken under light microscopy.

####  Scanning electron microscopy

SEM images of the fabricated MNPs were captured with a Zeiss LEO 1525 with a laser power of 5 kV. MNP samples were drop-cast onto a microscope slide being heated by a hotplate at 50 °C. When the sample was completely dried, a carbon tape covered mounting stub was pressed into the drop-cast to lift the particles off the microscope slide. For both MP and NPs, further images were taken, improving their dispersion prior to imaging. Storage medium was removed through centrifugation, and the particles were resuspended in 2-propanol and water for MPs and NPs, respectively. A drop containing the particles of each polymer was placed onto holey carbon film 300 mesh TEM grids (Product No. 931152, Merck). After the grids were dried, they were placed onto carbon tape covered SEM stubs. All samples were sputter coated with 15 nm chromium prior to imaging.

####  Raman microspectroscopy

Raman spectra of fabricated MNPs were obtained using a WiTech alpha300 RA S (Oxford Instruments, Abingdon, United Kingdom), equipped with a 532 nm diode laser at 30 mW laser power, in the CCD1 configuration. Samples were prepared by drop casting onto a CaF_2_ multispectral microscope slide. Spectra were captured at 100x magnification using a Zeiss ECC Epiplan-Neofluar HD DIC objective, over 20 accumulations at two seconds each. Raman spectra of each polymer and MNP type was matched against ca. 1400 S.T. Japan spectral database. Single MPs and MPFs were analysed, while NPs had to be pelleted to acquire a spectrum, due to the size constraints of the system. The hit quality index (HQI) was > 80 for all MNPs analysed, indicating a high similarity to the database spectra and chemical homogeneity.

#### Thermal desorption (TD)

##### Sample preparation

The MNPs were placed in 30 µL DMI sample inserts (GL Sciences Inc, Iruma, JP), precleaned by heating at 600 °C for 1 h using a Carbolite Gero muffle furnace (Carbolite Gero Ltd, Hope, UK). These inserts were placed into LINEX DMI Tapered Liners (GL Sciences Inc, Iruma, JP) precleaned in the same manner as the inserts. Liners were capped to limit potential sample contamination and placed onto the GCMS. A PAL-3 autosampler (Agilent Technologies, CA, USA) introduced the samples to an Optic-4 multimode (GL Sciences Inc, Iruma, JP) where the sample was thermally desorbed at 300 °C and injected onto the gas chromatography (GC) column. Sample analysis was performed using an Agilent 7890B GC with a LECO Pegasus BT 4D Time of flight mass spectrometer detector. Data acquisition was performed using LECO ChromaTOF software. Details of the instrument conditions are provided in Table 3.

**Table 3:** Instrument parameters for thermal desorption sample analysis

Inlet temperature	300 °C
GC conditions	
1st column	HP5MS (Agilent Technologies, CA, USA) 30 m x 0.25 mm, 0.25 μm i.d
2nd column	RXI17MS (RESTEK) 0.8 m x 0.25 mm, 0.25 μm i.d
Oven temperature profile	50 °C (2 min hold) → [10 °C/min] → 250 °C → [20 °C/min] → 300 °C (15 min hold)
I njection temperature	300 °C
Split flow	100:1
Carrier gas	Helium
Column flow	1.4mL/min
Modulation period	3s
MS conditions	
Emission current	1.0 mA
Ion source temperature	250 °C
Electron energy	70.0 eV
Transferline temperature	300 °C
Mass range	40–600 m/z
Scan rate	250 scans/s
Extraction frequency	28 kHz

##### Data processing

The collected data was processed using ChromaTOF software (LECO Corporation, MI, USA). An untargeted data processing method was used to locate all peaks within the sample with a signal to noise of > 50 eluting before 1800s, identifying those peaks using a built-in NIST mass spectral library. This produced a peak table of at least 700 identified peaks for each sample, peaks were then filtered to include only those with a spectral similarity of > 800. This reduced each sample to ~ 20–40 peaks. The mass spectra of identified peaks were checked for similarity with the library match and then assigned a chemical class. MNPs were prepared and analysed in duplicates, chemical compounds were ‘detected’ if they were found in both duplicates, these can be found in Table S.2, along with the chemical class of each compound.

#### Inductively coupled plasma mass spectrometry

To prepare water for metal analysis, 60 g of Chelex-100 ^tm^ resin was mixed with 2 L of Milli-Q water, which was covered in Parafilm and left to stir overnight in a plastic container. Chelex-100 ^tm^ resin was then removed by vacuum filtration with a 0.45 μm pore size nitrocellulose filter. pH of the final product was corrected to 7 using 1 M HCl and 1 M NaOH.

All glass and plasticware was washed with 20% Trace Metal grade nitric acid diluted in Chelex-100^tm^ treated water. 3 mg of MNPs was washed with 3 mL of 2% nitric acid solution in a tube vortexing for 24 h at 37 °C. MNPs were left to settle for 1 h, before being transferred to 1.5 mL tubes and centrifuged at 21,100 RCF for 2 h, three separate times to remove residual MNPs. 1 mL of the particle-free solution was transferred to a fresh set of acid washed 1.5 mL tubes.

Samples were spiked with Rh to a final concentration of 2 ug/L as an internal standard to monitor and correct for any changes in signal sensitivity. A multi-element solution was used to create calibration standards ranging from 0.01 to 100 ug/L. An MVX-7100 uL Workstation (Teledyne Cetac Technologies) autosampler was used for microflow sample introduction using a flow rate of 50 uL/minute. Samples were analysed using a Thermo Scientific iCAPQ inductively coupled plasma mass spectrometer (ICP-MS). Samples were split into 3, 0.3 mL aliquots each analysed over three measurement sessions on three different days. Procedural blanks were run alongside the samples and used to determine the LODs and LOQs. The final reported concentrations are the average +/- the standard deviation of the values determined over the three measurement sessions.

####  Cell culture

Transformed type 1 (TT1) alveolar epithelial cells were provided by T Tetley [39]. THP-1 monocytes were sourced from ATCC, USA. TT1s were cultured in hybridoma serum-free medium (HSFM) supplemented with 10% newborn calf serum (NCS), 2 mM L-glutamine and 1% Penicillin-Streptomycin. THP-1s were cultured in Roswell Park Memorial Institute-1640 (RPMI) supplemented with 10% foetal bovine serum (FBS), 10 mM HEPES buffer, 1 mM sodium pyruvate and 2mM L-glutamine. Both cell lines were cultured at in a humidified incubator at 37.5 °C and 5% CO_2_. TT1s were seeded at a density of 5.5 × 10^3^ cells/well in complete medium. THP-1s were seeded at a density of 1.5 × 10^4^ cells/well and differentiated into a macrophage-like phenotype using 100ng/ml of phorbol 12-myristate-13-acetate (PMA) for 48 h. Cells were allowed to rest for 24 h in complete medium after differentiation.

####  MNP leachate preparation and exposure

MNP leachates were assessed for toxicity in both TT1 and THP-1s. The leachates were prepared by resuspending each MNP in both types of medium containing 2% serum and incubated under normal culturing conditions, at 100 µg/mL for 24 h. This represents a common time point and dose for an in vitro MNP toxicity experiment [40–43]. Leachates were then centrifuged at 21,100 RCF for 45 min to remove the suspended MNPs. 200 µL of leachate was added to each well in triplicate, to expose the cells. Cells were then assayed for cellular metabolic activity after 24 h. Copper oxide (CuO) (< 50 nm particle size, 544868, Sigma Aldrich) was used as a positive control particle.

####  MTT assay

Cellular metabolic activity was assessed using an a 3-[4,5-dimethylthiazol-2-yl]-2,5 diphenyl tetrazolium bromide (MTT) assay, which is reduced into formazan by metabolically active cells (CT01, Sigma Aldrich). Leachate containing medium was removed and replaced with 100 µL of fresh 2% serum medium. 10 µl of MTT reagent was added to each well and allowed to incubate for 24 h under normal culturing conditions. Any formazan crystals were dissolved by the addition of 100 µl of isopropanol containing 0.04 N HCl. Plates were read at a test wavelength of 570 nm and a reference wavelength of 630 nm. Cellular metabolic activity was calculated as a percentage of the untreated controls.

####  Statistical analysis

GraphPad Prism was used for statistical analysis and generation of graphs. Two separate technical replicate MNP samples were used for TD and ICP-MS measurements. MTT analysis of MNP leachates were performed on three biological replicates. A non-normal distribution of data was assumed for low n-numbers. Therefore, a non-parametric Kruskal-Wallis test, alongside a post-hoc Dunn’s test, was used to compare independent groups of MNPs leachates and their effect on cellular metabolic activity. Untreated cells were used as a negative control, while CuO was used as a positive control. Standard deviation was used for displaying the data spread for graphical and tabular analysis.

## Results

The method described in this work uses the precipitation of bulk polymer solutions to produce a library of pristine micro/nanoplastic particles and fibres of a respirable size.

###  Physical characterization of fabricated MNPs

####  Morphology

**Fig. 1:**
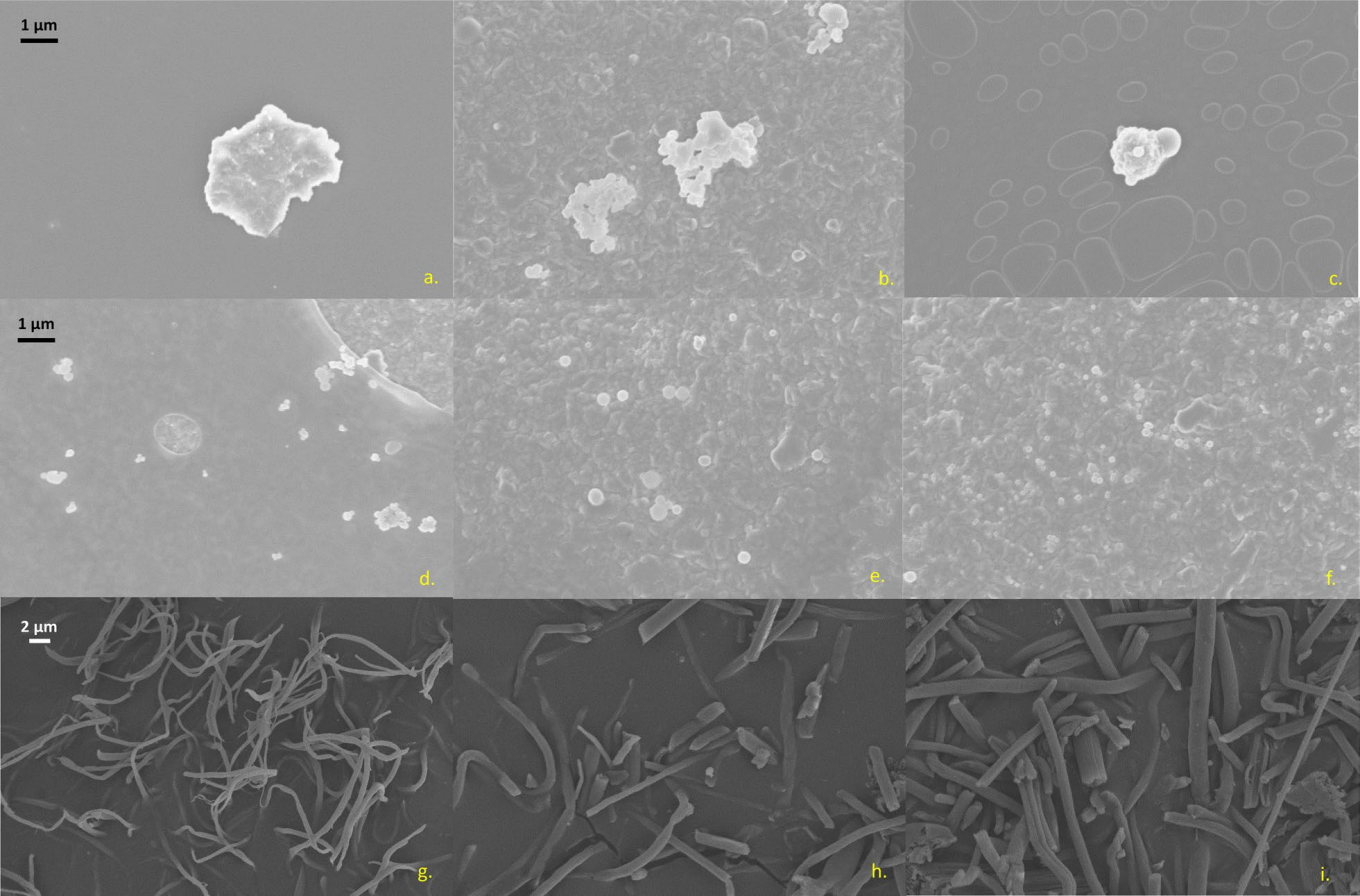
Scanning electron microscopy images of polyamide (**a**, **d**, **g**), polystyrene, (**d**, **e**, **f**) and polyethylene terephthalate (**c**, **f**, **i**) fabricated into micro (**a**–**c**), nano (**d**–**f**) and fibrous (**g**–**i**) plastics. Images of micro- and nanoplastics were taken at 50 K, while fibres were taken at 3 K magnification. The scale bars in the images of the first column apply to all images in their respective rows: (**a**–**c**), (**d**–**f**), and (**g**–**i**)

Discriminating individual particles in SEM images is challenging due to their tendency to aggregate when drying. To observe structural differences between the fabricated MNPs and minimize aggregation, we performed imaging on TEM grids. Fabricated MPs appear highly irregular (Fig. 1a–c), while NPs are more spherical (Fig. 1d–f). MPs have a much higher surface area than a typical spherical MPs would have, which may lead to an increased absorptive capacity and reaction area. More images of MP and NPs can be found in the Supplementary material (Figure S2-4). MPFs also present as slightly bent compared to how they look immediately after spinning, implying they exhibit some degree of flexibility (Fig. 1g–i) (Figure S1). Their uniform diameter also indicates a consistent electrospinning procedure.

####  Size distribution

**Fig. 2:**
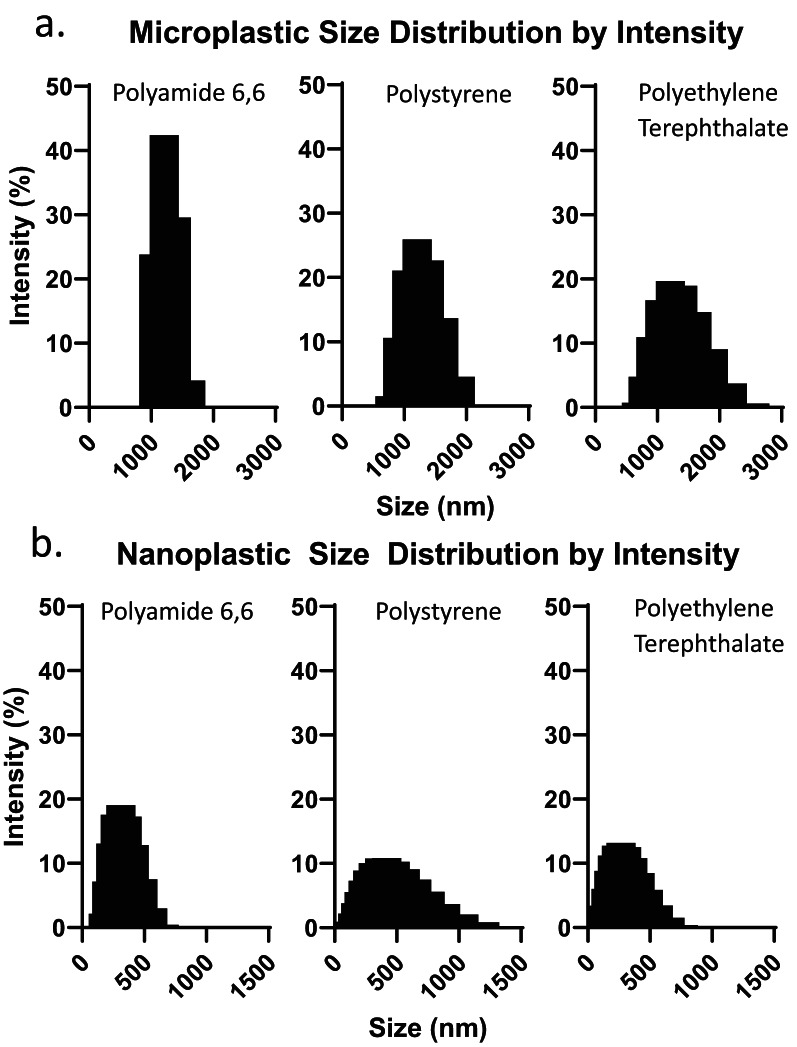
Micro (**a**) and nanoplastic (**b**) size distribution of polyamide 6,6, polystyrene, and polyethylene terephthalate as measured by dynamic light scattering on a Malvern Panalytical Zetasizer Pro. Measurements are reported as the intensity of light scattering (%) by the sample

DLS was used to investigate MP and NP size (Fig. 2). In ethanol, PA, PS, and PET MPs average size (z-average) was 1667, 1266, and 1371 nm, respectively. They have a polydispersity index of 0.3978, 0.2462, and 0.2117, respectively, indicating a moderate degree of polydispersity. In water, the PA, PS, and PET NPs average size was 309, 362 and 249 nm respectively. They exhibit a lower polydispersity index than the MPs at 0.1282, 0.1974, and 0.1692, respectively. Ethanol and water were determined to be the best dispersants for ensuring particle stability while also capturing the average size most representative of a single particle. Length, width, and aspect ratio of fabricated MPFs were calculating using ImageJ (Table 3). All measured fibres had a diameter under 3 μm, only 2% of PET and PA, and 8% of PS fibres had a length under 5 μm. All fibres had an aspect ratio > 3:1, classifying them as potentially respirable fibres [44] (Table 4).

**Table 4:** Fibre lengths of polyamide 6,6, polystyrene and polyethylene terephthalate, as produced by electrospinning and cryosectioning, measured using ImageJ

Polymer	Length (µm)	Width (µm)	Aspect ratio
Polyamide 6,6	12.06 ± 13.56	0.42 ± 0.122	28.71
Polystyrene	15.98 ± 13.56	1.00 ± 0.193	15.98
Polyethylene terephthalate	12.98 ± 8.12	1.02 ± 0.268	12.73

### Chemical characterization of fabricated MNPs

####  Zeta-potential

The zeta-potential of both MPs and NPs were performed in DI water and in phenol-free RPMI-1640 as a representative cell culture medium (CCM) with 2% serum (Table 5). As zeta-potential is dependent on the dispersant of the particles, CCM was used to model an in vitro scenario more accurately, while DI water was used as a baseline. In DI water, PA exhibits a zeta-potential close to neutral, while PS and PET are much lower, at around − 32 mV. In CCM, all particles have a zeta-potential of -12 to -16 mV, likely due to the formation of a corona formed around the particles due to the protein content of the serum.

**Table 5:** Zeta-potentials of fabricated micro and nanoplastics in water and cell culture medium (CCM) containing 2% foetal bovine serum

	Micro (mV)		Nano (mV)	
	CCM	Water	CCM	Water
Polyamide 6,6	− 13.000	5.423	− 15.440	− 5.900
Polystyrene	− 12.705	− 29.365	− 13.570	− 31.625
Polyethylene terephthalate	− 14.225	− 34.9	− 13.095	− 30.35

Zeta-potentials were measured using a folded capillary cell with a Malvern Panalytical Zetasizer Pro

####  Raman microspectroscopy

**Fig. 3:**
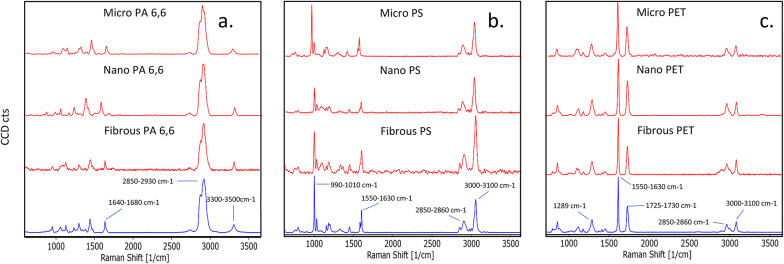
Raman spectra of polyamide 6,6 (PA) (**a**), polystyrene (PS) (**b**), and polyethylene terephthalate (PET) (**c**), measured using a 532 nm laser. Experimental spectra of the polymer shape (micro, nano, or fibrous), are displayed in red, while the reference spectra are displayed in blue. Key peaks are annotated with their corresponding wavelength

Raman microspectroscopy was used to confirm the chemical identity of fabricated plastics (Fig. 3). Key Raman absorption bands and their matching bonds are reported in Table 6 [45].

**Table 6:** Key Raman absorption bands and their matching chemical bonds for polyamide 6,6, polystyrene, and polyethylene terephthalate

Polymer	Key absorption bands	Chemical bond
Polyamide	1640–1680 cm^− 1^	Amide I (C = 0)
	2850–2930 cm^− 1^	Aliphatic C-H
	3300–3350 cm^− 1^	N-H
Polystyrene	990–1010 cm^− 1^	Aromatic C-C
	1550–1630 cm^− 1^	Aromatic C-C
	2850–2860 cm^− 1^	Aliphatic C-H
	3000–3100 cm^− 1^	Aromatic C-H
Polyethylene Terephthalate	1289 cm^− 1^	C-O
	1550–1630 cm^− 1^	Aromatic C-C
	1725–1730 cm^− 1^	C = O
	2850–2860 cm^− 1^	Aliphatic C-H
	3000–3100 cm^− 1^	Aromatic C-H

#### ICP-MS and TD measurements

ICP-MS (Table S1) and TD (Table S2) measurements revealed the presence of metal and organic contaminants associated with the fabricated MNPs. Commonly detected metals within the analysed samples were Al, Ti, V, Cr, Fe, Cu, Zn, Zr, Sr, Sn, and Ba. Some irregularities can be seen in the high levels of Mg observed in PET NPs at 1288.7 ± 173.0 ng/mg, Sb in PET MPs at 44.27 ± 5.04 ng/mg, and Fe and Cr in PA MPs at 180.4 ± 130.3 and 96.3 ± 74.9 Ti, Al and V are detected consistently across all MNPs, likely a result of the titanium alloy probe used for ultrasonication. Metals that were below the limit of quantification (LOQ), were reported as below quantification (BQ). A number of organic compounds were detected, with no one compound detected in all samples; commonly detected compounds were 1,3-benzenedicarboxylic acid (6/9) bis(2-ethylhexyl) ester, 1,2-epoxyundecane (4/9), and tetracosane (4/9). PA and PET have thermal decomposition temperatures above the inlet temperature of 300 °C, while PS starts decomposing at around 270 °C [46–48]. However, PS thermal decomposition products would primarily be aromatic hydrocarbons, such as styrene mono-, di-, trimers, toluene, and benzene [49]. The only detected aromatic hydrocarbons were 1,3-benzenedicarboxylic acid, a plasticizer detected in several samples, 1-propanamine, 3-dibenzo[b, e]thiepin-11(6 H)-ylidene-N, N-dimethyl-, S-oxide, detected only in PS MPs but also PA MPs and PET MPFs, and benzoyl isothiocyanate, detected only in PS MPs, but is nitrogen-containing and not part of the polymer chain. We can be confident the detected compounds are the products of contaminants and not of the polymer chains.

###  MNP leachates had no effect on cellular metabolic activity

**Fig. 4:**
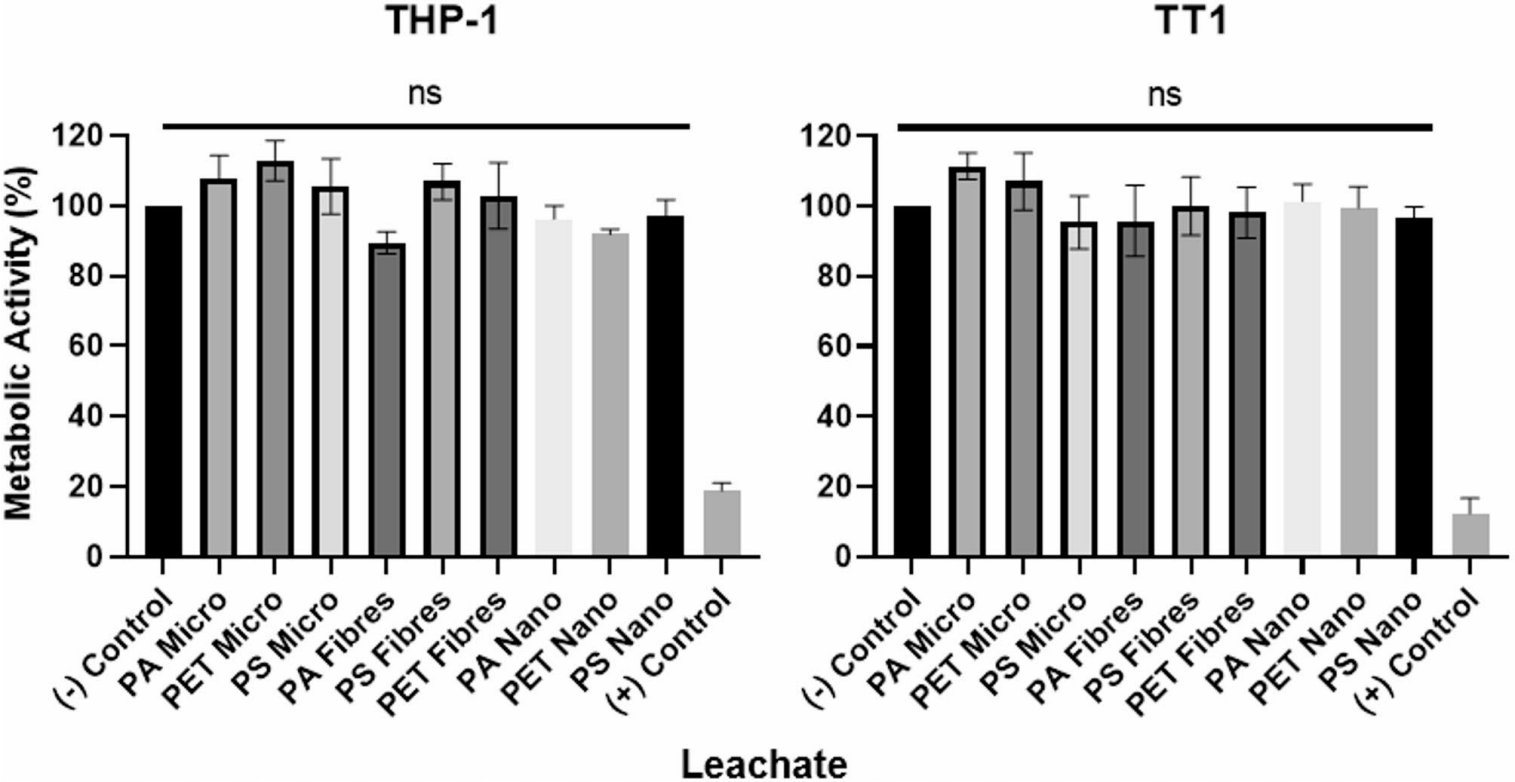
Effects of polyamide (PA), polystyrene (PS), and polyethylene terephthalate (PET) MNP leachates on the metabolic activity of TT1 epithelial cells and THP-1 macrophages. Cells were exposed to a leachate representative of a 100 µg/mL dose for 24 h. Cellular metabolic activity is represented as the percentage change from of MNPs the untreated control. A Kruskal-Wallis with a post hoc Dunn’s test was used to determine statistical significance relative to untreated cells, (*n* = 3). Copper oxide (CuO) was used as a positive control

Despite the washing steps, there remains a potential for chemical additives from the plastic feedstocks or contaminants from the fabrication methods to leach into the exposure media and contribute to toxicity not inherent to the MNPs. 100 µg/mL of MNPs were incubated in either RPMI or HSFM for 24 h and exposed to their respective cell type. None of the MNP leachates had an effect on the cellular metabolic activity of either cell type (Fig. 4).

## Discussion

In this work, PA, PS and PET MNPs have been fabricated in a size range that is suitable for studying the biological effects of MNPs in the lung. Unlike previous studies, which may have only covered two or three types of MNPs, our MNP library contains MNPs of two shapes, two sizes, and three different polymers. These can be used as a toolkit for investigating their toxicological hazard and provide complementary MNPs to existing fabrication methods. Our MNPs cover three polymers that are highly represented in air samples, as both fibres and irregularly shaped particles [4, 14, 50, 51]. PA, PS, and PET are among the most commonly detected environmental polymers, alongside PP and PE, with PET being the most common of the three, followed by PS and PA, depending on the environment sampled [3, 4, 8, 14, 16, 17, 51]. Their highly irregular shape also provides a more accurate representation of a typical environmental MNP [52, 53]. PP and PE were excluded from this study as they are not easily soluble at room temperature and are difficult to electrospin [29, 33]. It should be noted that a limitation with the current method is the production of grams of MNPs, due to the milligram concentrations of the solutions used. However, milligram quantities are typically sufficient for toxicological studies, as most experiments require only small amounts of material to assess biological responses.

We have fabricated MNP fibres with dimensions capable of penetrating the deep lung [44, 54]. Despite fibres being a common shape for atmospheric microplastics, and there being evidence of their release, particularly from clothing, few papers have investigated the hazard they may pose to human health if inhaled [20, 55, 56]. Our fabricated MPFs provide a suitable test material to do so, as fibrous materials with a diameter of < 3 μm and length > 5 μm are of suspected of causing chronic inflammation, penetration into the pleural cavity, and carcinogenesis in the lung [57, 58]. It should be noted that while PET and PA fibres are common, PS fibres are not, they were produced purely to help answer the question of shape, as PS has been widely used in toxicity assays. The method also allows for the fabrication of different sized fibres by changing the cutting size on the cryotome, as fibre length and rigidity that have been suggested as critical factors in their toxicity [59–61]. These fibres more closely mimic fibrils produced from larger clothing fibres due to abrasion and wear [20]. Despite the difference in diameter between the PA and PET/PS MPFs (~ 0.5 μm vs. ~ 1 μm), few studies have investigated the toxicological dependence of fibre diameter at these sizes. While this may result in differences in ‘real world’ scenarios, such as their bio-persistence and deposition within the lung, we believe they are suitable comparators for preliminary toxicological experiments.

While the fabricated MPs are non-spherical, as environmental MNPs tend to be, this becomes a limitation when it comes to characterizing the size of MNPs using DLS. DLS assumes a perfectly spherical particle in suspension, in the dispersant of choice for the measurement. When the particles are non-spherical, it reports the diameter of a theoretical sphere which most accurately describes the particle being measured [62]. Additionally, MNP stability in the dispersant is key, MPs were measured in ethanol due to poor stability in aqueous solutions, whereas NPs were more stable in water. Although both PS and PET MNPs exhibit a zeta-potential greater than − 30 mV in water, which is considered the threshold for a stable dispersion [63–65], in practice, MNPs of both polymers adhere quickly to themselves and to their containers. While neither the aggregation kinetics nor influence of the dispersant are explored herein, agglomeration of MNPs is likely driven by hydrophobic interactions. Even in their respective dispersants, MNPs may still agglomerate, compromising the accuracy of DLS measurements by increasing apparent particle size, altering the size distribution and polydispersity. To ameliorate this, ultrasonication was performed prior to DLS measurements to disperse the MNPs, however agglomeration over the course of the measurements may still occur. The z-average, or average hydrodynamic size reported here and in many other studies is dependent on the dispersant and stability of the particle being measured, the latter of which should take priority, as the measurement of individual particles provides a more accurate representation of the average particle size.

It should also be noted that FBS was used as a surfactant for NPs in this methodology. Its addition is essential for maintaining NP stability during the fabrication process and improves the quality of DLS measurements. The use of FBS in in vitro studies may significantly influence the toxicity of particulates by modifying their cellular interactions through biomolecule coronation. This corona may alter particle properties, enhance uptake, and can either attenuate or exacerbate cytotoxic effects depending on the particle type and mechanism of toxicity [66, 67]. While FBS is not entirely representative of the alveolar region, the pulmonary surfactant contains a number of proteins and lipids that coronates particulates following alveolar deposition [68, 69]. A different biocompatible surfactant may also be used to stabilize NPs depending on the needs of the researcher.

The shortcomings of DLS for MNP analysis can be supplemented with SEM imaging which allows high resolution imaging of individual particles and hence inform DLS size data by visualising particle morphology. However, MNPs aggregate while drying due to their strong hydrophobic interactions, this makes particles difficult to discriminate while imaging, especially when they exhibit irregular shapes. These issues can be visualized in two recent publications attempting to image their fabricated MNPs [23, 29]. Our SEM images reveal that MPs exhibit a much more irregular shape than the NPs, and also a higher PDI. The turning and movement of irregular particles may also cause changes in light scattering, altering the diffraction pattern. As DLS measurements assume sphericity, measurements of irregular particles may vary more as a result of inconsistent assumptions regarding the hydrodynamic size, increasing the PDI. Furthermore, as DLS is not a suitable method to measure particles with a high aspect ratio, the zeta potential of MPFs was not analysed. It is assumed that their zeta potential is similar to their micro/nano counterparts, as they themselves show only a few mV of difference.

Chemical analysis of fabricated MNPs was conducted to determine polymer identity, assess metal content, organic chemicals, and zeta-potential. The chemical make-up of the MNPs comes down to the stock polymer itself, the fabrication process, and the difference in chemical structure which may affect their affinity to certain exogenous compounds. Leachate studies on the MNPs showed that the presence of any adsorbed chemicals did not affect cell metabolic activity in either TT1 epithelial cells or THP-1 macrophages after 24 h. The importance of any organic and metal contamination should still be considered depending on the experimental setup, but not necessarily as a toxic component of the MNPs. We encourage researchers to verify this by performing incubations over a time period appropriate to their experiment. Furthermore, aliquoting NPs at a working concentration prior to storage is recommended to reduce the potential effect of freeze-thawing on polymer stability and structure.

Airborne MNPs are rarely spherical, and our new test material will serve as a complementary set of MNPs for toxicologists. Using the proposed fabrication method, a stock of MNPs can be built up and aliquoted to minimize inter-batch variation, maintain repeatability, and allow for data comparisons between experiments. The properties characterized herein reflect those of a pristine material that may not fully capture environmental weathering conditions. Future research can use this as a base material and explore different states of weathering alongside pristine MNPs. This is an adaptable material that can be loaded or co-exposed with other chemicals, weathered, and dyed for a number of experimental designs. We hope this will be an accessible way to advance research beyond single polymer/size/shape studies, to further understand the differential biological effects of different MNPs. We are already in collaboration with several research groups and are open to providing material or assisting with the fabrication process. Further analysis on the MNPs could be conducted to determine crystallinity, density, surface area, and particle surface valence.

## Supplementary Information

Supplementary material 1.

## Data Availability

No datasets were generated or analysed during the current study.

## Abbreviations

CuO: Copper oxide
DI: Deionized water
DLS: Dynamic light scattering
FA: Formic acid
FBS: Foetal bovine serum
GC: Gas chromatography
HCL: Hydrochloric acid
HFIP: 1,1,1,3,3,3-hexfluoro-2-isopropanol
HSFM: Hybridoma serum-free medium
ICP-MS: Inductively-coupled plasma mass spectrometry
LOD: Limit of detection
LOQ: Limit of quantification
MNP: Micro/nanoplastics
MP: Microplastics
MPF: Microplastic fibres
MTT: 3-(4,5-dimethylthiazol-2-yl)-2,5-diphenyltetrazolium bromide
NaOH: Sodium hydroxide
NCS: Newborn calf serum
NP: Nanoplastic
OCT: Optimal cutting temperature
PA: Polyamide 6,6
PET: Polyethylene terephthalate
PM: Particulate matter
PMA: Phorbol 12-myristate 13-acetate
PS: Polystyrene
PTFE: Polytetrafluoroethylene
Py-GC-MS: Pyrolysis-gas chromatography- mass spectrometry
RCF: Relative centrifugal force
RI: Refractive index
RPM: rotations per minute
RPMI: Roswell Park Memorial Institute
SEM: Scanning electron microscopy
SS: Stainless steel
TD: Thermal desorption
THF: Tetrahydrofuran
TT1: Transformed type 1
