# Supplementary material for: Fabrication of microplastic and nanoplastic particles and fibres for use in pulmonary toxicity studies
Source: Part Fibre Toxicol. 2025 Nov 6;22:30. doi: 10.1186/s12989-025-00641-w (PMC12590796; doi:10.1186/s12989-025-00641-w)
Supplement: Supplementary file 1 — Supplementary material 1. [file 12989_2025_641_MOESM1_ESM.docx]

| Table S1. ICP-MS metal analysis of fabricated micro, nano, and fibrous plastics. Data is presented as ng of metal /mg of plastic material, BQ stands for ‘below quantification’ which was defined as the LOQ for each measurement. The standard deviation was calculated from replicate measurements of the same samples. Two samples were analysed and averaged together to obtain the final value. | | | | | | | | | | | | |
| --- | --- | --- | --- | --- | --- | --- | --- | --- | --- | --- | --- | --- |
| ng of metal/mg of plastic | **Polyamide Microparticles** | **Polystyrene Microparticles** | **Polyethylene Terephthalate Microparticles** | **Polyamide Nanoparticles** | **Polystyrene Nanoparticles** | **Polyethylene Terephthalate Nanoparticles** | **Polyamide Fibres** | **Polystyrene Fibres** | **Polyethylene Terephthalate Fibres** | **Isopropanol** | **Procedural blanks** |  |
| Mg | 17.6 ± 6.0 | BQ | BQ | BQ | 120.3 ± 91.0 | 1288.7 ± 173.0 | BQ | BQ | BQ | BQ | BQ |  |
| Al | 49.0 ± 7.1 | 39.7 ± 6.6 | 35.7 ± 6.3 | 34.3 ± 8.1 | 36.7 ± 6.3 | 38.7 ± 6.1 | 76.5 ± 17.2 | 67.0 ± 9.0 | 174.6 ± 33.4 | 39.1 ± 5.8 | 26.2 ± 4.5 |  |
| Ti | 0.79 ± 0.24 | 1.56 ± 0.53 | 0.60 ± 0.09 | 0.85 ± 0.14 | 1.23 ± 0.47 | 2.81 ± 0.35 | 1.17 ± 0.57 | 0.87 ± 0.10 | BQ | BQ | BQ |  |
| V | 0.73 ± 0.54 | 0.35 ± 0.19 | BQ | 0.10 ± 0.02 | 0.09 ± 0.02 | 0.26 ± 0.17 | 0.45 ± 0.07 | 0.78 ± 0.08 | 14.84 ± 1.36 | BQ | BQ |  |
| Cr | 96.3 ± 74.9 | 45.4 ± 28.8 | 1.76 ± 0.77 | 3.58 ± 0.66 | 4.53 ± 0.97 | 31.1 ± 29.8 | 1.53 ± 0.17 | 3.19 ± 0.24 | 0.51 ± 0.05 | 0.84 ± 0.42 | 0.99 ± 0.60 |  |
| Mn | 4.32 ± 3.38 | 1.02 ± 0.44 | BQ | BQ | 0.60 ± 0.26 | 2.88 ± 0.64 | 0.70 ± 0.35 | 0.99 ± 0.26 | BQ | BQ | BQ |  |
| Fe | 180.4 ± 130.3 | 69.4 ± 34.1 | 9.62 ± 1.98 | 13.6 ± 3.3 | 24.4 ± 6.9 | 67.6 ± 42.0 | 21.0 ± 3.2 | 23.1 ± 3.9 | 6.8 ± 1.4 | BQ | BQ |  |
| Co | 0.73 ± 0.54 | 0.25 ± 0.13 | BQ | BQ | BQ | BQ | BQ | BQ | BQ | BQ | BQ |  |
| Ni | 78.0 ± 32.5 | 14.3 ± 8.9 | BQ | BQ | BQ | BQ | 28.3 ± 3.3 | 20.3 ± 2.7 | 6.95 ± 0.77 | BQ | BQ |  |
| Cu | 5.48 ± 3.36 | 2.17 ± 0.37 | BQ | BQ | 1.84 ± 0.32 | 2.70 ± 1.55 | 2.14 ± 0.2 | 14.3 ± 3.6 | 31.5 ± 5.1 | BQ | BQ |  |
| Zn | 63.8 ± 44.4 | 73.2 ± 34.9 | 28.5 ± 12.6 | 43.3 ± 25.3 | 34.7 ± 4.4 | 89.8 ± 10.4 | 48.1 ± 6.1 | 18.8 ± 4.1 | 49.5 ± 8.6 | 49.9 ± 24.3 | 91.2 ± 67.2 |  |
| As | BQ | BQ | BQ | BQ | BQ | BQ | 0.09 ± 0.01 | BQ | BQ | BQ | BQ |  |
| Rb | BQ | BQ | BQ | BQ | 1.04 ± 0.35 | 6.17 ± 1.33 | BQ | BQ | BQ | BQ | BQ |  |
| Sr | 0.60 ± 0.31 | 0.57 ± 0.07 | 0.24 ± 0.03 | BQ | 1.63 ± 0.87 | 6.10 ± 0.66 | 0.25 ± 0.07 | 0.23 ± 0.04 | BQ | 0.19 ± 0.03 | 0.15 ± 0.01 |  |
| Zr | 0.56 ± 0.08 | 0.54 ± 0.11 | 0.44 ± 0.06 | 0.47 ± 0.11 | 0.41 ± 0.09 | 0.21 ± 0.03 | 0.51 ± 0.06 | 0.59 ± 0.1 | 0.72 ± 0.09 | 0.61 ± 0.05 | 0.25 ± 0.14 |  |
| Mo | 0.85 ± 0.53 | 0.38 ± 0.17 | BQ |  | 0.14 ± 0.03 | 0.34 ± 0.21 | 0.07 ± 0.01 | 0.08 ± 0.01 | 0.08 ± 0.01 | BQ | BQ |  |
| Ag | 0.05 ± 0.01 | BQ | BQ | BQ | 0.08 ± 0.02 | 0.08 ± 0.01 | BQ | 0.06 ± 0.01 | 0.08 ± 0.01 | BQ | BQ |  |
| Sn | 0.88 ± 0.19 | 0.84 ± 0.21 | 1.04 ± 0.43 | 0.55 ± 0.10 | 0.54 ± 0.06 | 0.57 ± 0.08 | 82.3 ± 8.8 | 5.03 ± 0.77 | 114.0 ± 10.6 | 0.92 ± 0.4 7 | 0.52 ± 0.05 |  |
| Sb | 1.02 ± 0.67 | 0.42 ± 0.09 | 44.27 ± 5.04 | BQ | BQ | 4.04 ± 0.46 | BQ | 0.21 ± 0.04 | 22.9 ± 2.4 | BQ | BQ |  |
| Ba | 0.73 ± 0.32 | 0.64 ± 0.17 | 0.58 ± 0.15 | BQ | 1.37 ± 0.62 | 2.95 ± 0.33 | 0.39 ± 0.01 | 1.51 ± 0.62 | 0.75 ± 0.20 | BQ | BQ |  |
| La | 0.05 ± 0.01 | BQ | BQ | BQ | 0.05 ± 0.01 | 0.06 ± 0.01 | 0.06 ± 0.01 | 0.08 ± 0.02 | 0.05 ± 0.04 | BQ | 0.12 ± 0.01 |  |
| Pt | BQ | BQ | 0.25 ± 0.07 | BQ | BQ | BQ | BQ | 0.80 ± 0.19 | 0.20 ± 0.03 | BQ | BQ |  |
| Pb | 1.49 ± 0.42 | 0.58 ± 0.10 | 3.64 ± 0.57 | 0.48 ± 0.09 | 0.97 ± 0.31 | 0.58 ± 0.08 | 0.57 ± 0.06 | 0.78 ± 0.11 | 5.90 ± 1.08 | 0.47 ± 0.06 | 0.34 ± 0.03 |  |
| Bi | 0.20 ± 0.09 | BQ | BQ | BQ | BQ | BQ | BQ | BQ | BQ | BQ | BQ |  |

Elements not detected in any of the MNP samples were: Sc, Ga, Ge, Ce, Nd, Sm, Eu, Gd, Tb, Dy, Ho, Er, Tm, Yb, Lu, Hf, Ta, W, Re, Hg, TL, Th, and U.

| Table S2. Thermal desorption analysis of fabricated micro/nanoplastics by polymer type. Green indicates a positive detection in both replicate samples. Chemical compounds and their class are displayed in the two left hand columns. | | | | | | | | | | |
| --- | --- | --- | --- | --- | --- | --- | --- | --- | --- | --- |
|  | | **Micro** | | | **Nano** | | | **Fibre** | | |
| Chemical compound | **Chemical class** | **PA** | **PS** | **PET** | **PA** | **PS** | **PET** | **PA** | **PS** | **PET** |
| 1,2-Epoxyundecane | Epoxide |  |  |  |  |  |  |  |  |  |
| 1,3-Benzenedicarboxylic acid,  bis(2-ethylhexyl) ester | Ester |  |  |  |  |  |  |  |  |  |
| 13-Methyltetradecanal | Aldehyde |  |  |  |  |  |  |  |  |  |
| 1-Docosanol, acetate | Ester |  |  |  |  |  |  |  |  |  |
| 1-Docosene | Alkene |  |  |  |  |  |  |  |  |  |
| 1-Dodecanol, 2-octyl- | Alcohol |  |  |  |  |  |  |  |  |  |
| 1-Dodecene | Alkene |  |  |  |  |  |  |  |  |  |
| 1H-Pyrrole-2,5-dione | Imide |  |  |  |  |  |  |  |  |  |
| 1-Pentadecene | Alkene |  |  |  |  |  |  |  |  |  |
| 1-Propanamine, 3-  dibenzo[b,e]thiepin-11(6H)-  ylidene-N,N-dimethyl-, S-oxide | Amine (with a thiepin and sulfoxide functional group) |  |  |  |  |  |  |  |  |  |
| 2-Tridecen-1-ol, (E)- | Alkene, Alcohol |  |  |  |  |  |  |  |  |  |
| 3-Eicosene, (E)- | Alkene |  |  |  |  |  |  |  |  |  |
| 4-Trifluoroacetoxyhexadecane | Ester |  |  |  |  |  |  |  |  |  |
| Benzoyl isothiocyanate | Isothiocyanate |  |  |  |  |  |  |  |  |  |
| Bis(2-ethylhexyl) phthalate | Ester (Phthalate) |  |  |  |  |  |  |  |  |  |
| Cholesta-3,5-diene | Steroid, Diene |  |  |  |  |  |  |  |  |  |
| Cholesterol, chlorodifluoroacetate | Steroid, Ester |  |  |  |  |  |  |  |  |  |
| Cyclopentanone | Ketone |  |  |  |  |  |  |  |  |  |
| Decane | Alkane |  |  |  |  |  |  |  |  |  |
| Docosane | Alkane |  |  |  |  |  |  |  |  |  |
| Dodecanal | Aldehyde |  |  |  |  |  |  |  |  |  |
| Dodecanamide | Amide |  |  |  |  |  |  |  |  |  |
| Dodecane | Alkane |  |  |  |  |  |  |  |  |  |
| Eicosanal- | Aldehyde |  |  |  |  |  |  |  |  |  |
| Heneicosane | Alkane |  |  |  |  |  |  |  |  |  |
| Heneicosanol | Alcohol |  |  |  |  |  |  |  |  |  |
| Heptadecanal | Aldehyde |  |  |  |  |  |  |  |  |  |
| Heptadecane | Alkane |  |  |  |  |  |  |  |  |  |
| Hexadecane | Alkane |  |  |  |  |  |  |  |  |  |
| Hexane, 1,6-diisocyanato- | Isocyanate |  |  |  |  |  |  |  |  |  |
| n-Heptadecanol-1 | Alcohol |  |  |  |  |  |  |  |  |  |
| n-Hexadecanoic acid | Carboxylic Acid (Fatty Acid) |  |  |  |  |  |  |  |  |  |
| n-Nonadecanol-1 | Alcohol |  |  |  |  |  |  |  |  |  |
| Nonadecane | Alkane |  |  |  |  |  |  |  |  |  |
| Octacosane | Alkane |  |  |  |  |  |  |  |  |  |
| Octadecanal | Aldehyde |  |  |  |  |  |  |  |  |  |
| Oxirane, tetradecyl- | Epoxide |  |  |  |  |  |  |  |  |  |
| Pentacosane | Alkane |  |  |  |  |  |  |  |  |  |
| Pentadecane | Alkane |  |  |  |  |  |  |  |  |  |
| Pentadecanoic acid | Carboxylic Acid (Fatty Acid) |  |  |  |  |  |  |  |  |  |
| RMDI, Cis,trans- | Isocyanate |  |  |  |  |  |  |  |  |  |
| RMDI, trans,trans- | Isocyanate |  |  |  |  |  |  |  |  |  |
| Tetracosane | Alkane |  |  |  |  |  |  |  |  |  |
| Tetradecane | Alkane |  |  |  |  |  |  |  |  |  |
| Tetradecyl trifluoroacetate | Ester |  |  |  |  |  |  |  |  |  |
| Tricosane | Alkane |  |  |  |  |  |  |  |  |  |
| Tridecane | Alkane |  |  |  |  |  |  |  |  |  |
| Undecanal | Aldehyde |  |  |  |  |  |  |  |  |  |


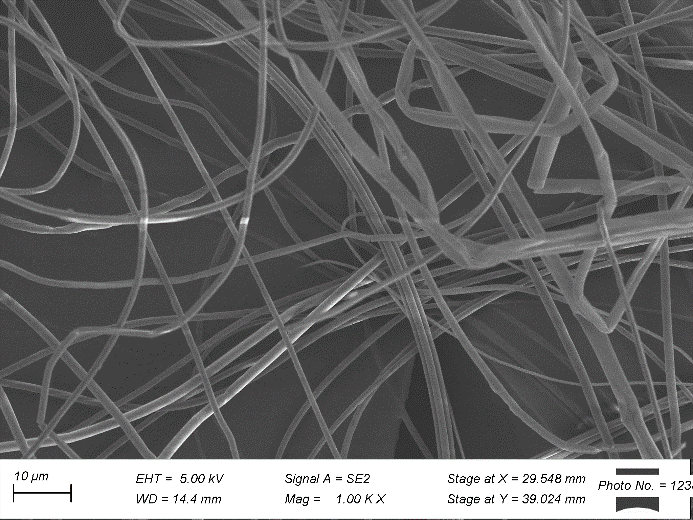

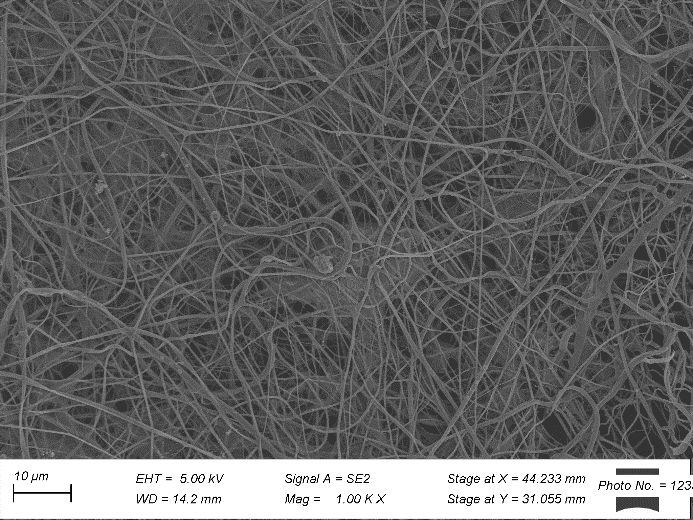


10 µm


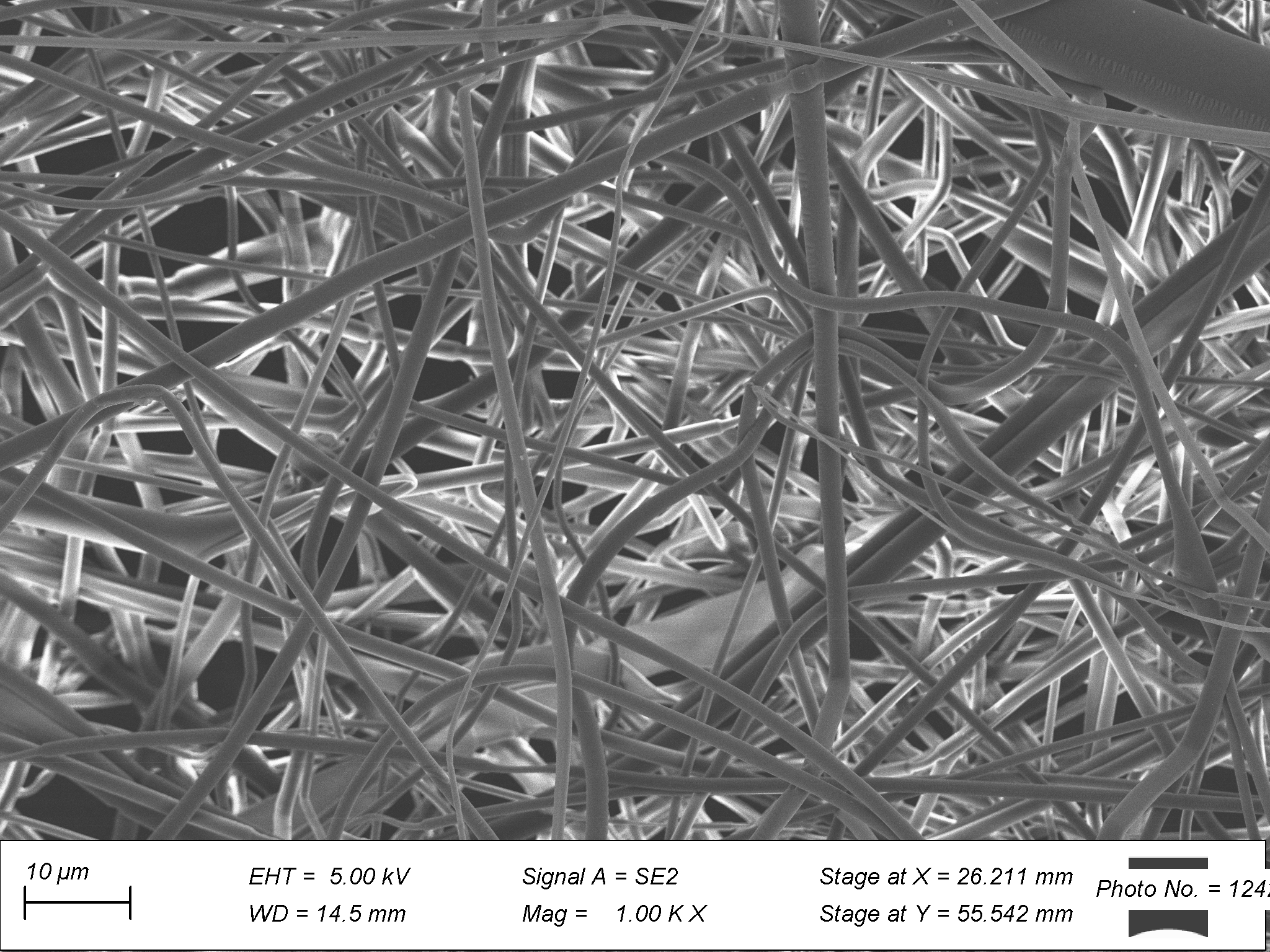


Figure S1. Uncut electrospun fibres of (a.) polyamide, (b.) polystyrene, and (c.) polyethylene terephthalate. Images were taken at 1,000 times magnification.









2078 nm

1352 nm

5320 nm

2668 nm

**2 µm**

**10 µm**

Figure S2. Scanning electron microscopy images of fabricated polyamide (a,d), polystyrene (b,e), and polyethylene terephthalate (f,c) **microplastics**. Images were taken at 5k (a-c) and 30k (d-f) magnification. The scale bars in the images of the first column apply to all images in their respective rows: (a–c) and (d–f). Example particle sizes can be seen in red.

f.

e.

d.

c.

b.

a.

3471 nm

2530 nm

1049 nm

2220 nm

1665 nm









1542 nm

1331 nm

5133 nm







`

**10 µm**

a.

b.

c.









**2 µm**

d.

e.

f.









**1 µm**

316 nm

179 nm

194 nm

316 nm

159 nm

212 nm

243 nm

639 nm

h.

302 nm

g.

i.

Figure S3. Scanning electron microscopy images of fabricated polyamide (a,d,g), polystyrene (b,e,h), and polyethylene terephthalate (f,c,i) **nanoplastics**. Images were taken at 5k (a-c), 30k (d-f) and 50k (g-i) magnification. The scale bars in the images of the first column apply to all images in their respective rows: (a–c), (d–f), and (g–i). Example particle sizes can be seen in red.
















Figure S4. Scanning electron microscopy images of polyamide, (a,d), polystyrene (b,e), and polyethylene terephthalate (c,f) fabricated into micro (a-c), nano (d-f) plastics dried on carbon tape. Images were taken at 30K and 200K magnification, respectively. The scale bars in the images of the first column apply to all images in their respective rows: (a–c) and (d–f).

a.

b.

c.

d.

e.

f.

**2 µm**

**200 nm**
